# TRASH OR TREASURE?: Putting Coal Combustion Waste to Work

**DOI:** 10.1289/ehp.117-a490

**Published:** 2009-11

**Authors:** David J. Tenenbaum

**Affiliations:** **David J. Tenenbaum**, feature writer at The Why Files, won the 2002 science writing award from the American Association for the Advancement of Science

Even as public debate rages over the question of whether coal should continue to provide the majority of U.S. electric power needs, the U.S. Energy Information Administration predicts in *International Energy Outlook 2009* that, absent new policies to the contrary, the United States—along with China and India—is expected to account for 88% of the projected net increase in coal consumption between 2006 and 2030. Meanwhile, coal combustion waste (CCW)—the noncombustible remains from coal burning—continues to pile up at the rate of about 131 million tons per year in the United States alone, and electric utilities are looking to recycle a larger proportion of this material.

The American Coal Ash Association (ACAA) reported in its latest *Coal Combustion Product Production & Use Survey Results* that 43% of all CCW produced in the United States in 2007 was devoted to what are termed “beneficial uses.” The state-defined “beneficial use” designation means a waste product is used in the manufacture of or as a replacement for another product. Waste products granted a state “beneficial use” determination are exempt from solid waste regulations governing their disposal.

An estimated 23% of the total CCW produced in the United States each year—more than 30 million tons—is used in construction products, primarily concrete and wallboard, but also clinker (raw material for making portland cement), roofing granules, saggregate for paving materials, and asphalt filler. In 2007, according to the ACAA, 14.5 million tons of CCW was used in concrete, another 5.0 million tons was used as clinker, and 8.3 million tons was used in gypsum wallboard, which is the standard interior wall material used in the United States. (In *EHP*’s exploration of the use of CCW in building products, the preponderance of studies and statistics focused on the situation in the United States.)

**Why does cement manufacturing produce so much CO_2_?**

Portland cement is made primarily from limestone and clay, which are heated in cement kilns at temperatures of about 2,700°F. Through a chemical process known as calcination, the raw ingredients take on the properties that make cement cement, including the ability to solidify and strengthen when mixed with water. The calcined raw materials form pebbles of “clinker” that are ground into portland cement. Fly ash does not depend on heating for its cementitous properties, so when it is used to partially replace portland cement in a concrete mix, producers save energy.

According to the Portland Cement Association, about 60% of the CO_2_ produced during cement production is a by-product of calcination. The rest is a product of the fuels used to heat the kiln—often coal. Because calcination is a necessary step in cement formation, other parts of the process must be modified if cement production is to reduce its energy use and CO_2_ output.

Other “beneficial uses” include applications that critics say are closer to unregulated dumping than to recycling. For example, about 6.7 million tons of CCW is used to fill abandoned mines, often as a measure to neutralize the acidic liquid that can drain from these sites into nearby waters. Another 10.6 million tons is used for structural fills and embankments. Under the “beneficial use” designation, these applications are exempt from safeguards that, depending on state law, may be required of disposed waste, such as the use of liners to prevent leaching of potentially toxic metals into ground and surface waters. This raises the potential for serious environmental consequences.

In *Environmental Concerns and Impacts of Power Plant Waste Placement in Mines*, a 2004 report on minefilling written for the U.S. Office of Surface Mining Reclamation and Enforcement, hydrogeologist Charles Norris reported that “ground waters and surface waters are being degraded by [minefilling]. Data from designated ash monitoring points show rises in the concentrations of total dissolved solids, sulfate, manganese, iron, boron, and a variety of trace heavy metals in these waters that significantly exceed baseline concentrations. . . . The data raise fundamental questions about the adequacy of safeguards in permits authorizing ash placement in coal mines and the assertions that alkaline coal ashes are inherently reliable and safe materials for preventing acid drainage or remediating abandoned mined lands.”

Given the potential for heavy metal contamination, questions have arisen about the advisability of adding CCW to construction products that will be used in roads, bridges, even homes. Do these types of “beneficial uses” also potentially expose people to toxic materials?

## CCW in Construction Materials

Many of the construction-related uses of CCW involve materials traditionally made using energy-intensive processes that release large amounts of greenhouse gases. For instance, the cement industry creates about 5% of global carbon dioxide (CO_2_) emissions; in the United States, producing a metric ton of portland cement releases, on average, an estimated 0.95 metric ton of CO_2_ equivalent [for more information on cement manufacturing, see sidebar at left]. Craig Benson, Wisconsin Distinguished Professor of civil and environmental engineering and geological engineering at the University of Wisconsin–Madison and codirector of the University of New Hampshire–based Recycled Materials Resource Center, calculates that each year in the United States recycling CCW saves about 160 trillion BTUs of energy (about the amount of energy used by 1.7 million households), 11 million tons of CO_2_ equivalent (comparable to the average annual emissions of more than 1.8 million passenger vehicles), and 32 billion gallons of water.

About 71.1 million tons, or 55%, of the CCW produced each year is fly ash, a fine material that is captured after combustion in filters or electrostatic precipitators. Fly ash is composed of microscopic spheres containing largely silica, iron, aluminum, and calcium; the biggest current construction-related use of fly ash is to replace portland cement, which binds the sand and gravel in concrete. Fly ash has different characteristics depending on the chemical content of the coal from which it derives. Broadly speaking, lignite and subbituminous coals produce Class C fly ash, which has self-cementing properties, whereas anthracite and bituminous coals produce Class F fly ash, which typically must be mixed with water and a cementing agent in order to harden.

Nationally, 8–12% of the binder in concrete is fly ash, says John Sager, who coordinates the Coal Combustion Products Partnership, an initiative of the U.S. Environmental Protection Agency (EPA) that promotes recycling of CCW. David Goss, the former executive director of the ACAA, says concrete floors fortified with fly ash are “highly polished, attractive, have high wearability, and eliminate the need for tile.” Fly ash can reduce alkali silica reactivity, a chemical reaction that can cause extensive cracking of concrete made with certain types of aggregate, adds Steven Kosmatka, vice president of research and technology services at the Portland Cement Association (PCA).

“Often the reaction is ‘I don’t want garbage—waste—hidden in my concrete,’” says Kosmatka. “But when people hear that fly ash contributes to strength development, can help improve durability, help with economics, reduce heat generation during setting, reduce permeability to keep chlorides away from steel reinforcement, and prevent corrosion, suddenly people don’t think of it as waste anymore—they think of it as a substance with a positive impact on the ultimate product.”

Fly ash can also stabilize soil beneath a highway. Benson says fly ash mixed into the upper 300 cm of soil “sets up like lean concrete and creates a really good working platform” that can replace the 1-m layer of crushed rock that is typically used beneath major highways. “We avoid the other 700 cm of fill and eliminate all the energy and emissions associated with excavating and crushing this rock,” says Benson.

Fly ash constitutes 50–85% of a wood replacement called LifeTime Lumber, produced by LifeTime Composites of Carlsbad, California. The material, used for decking and fencing, is inedible to termites and does not support mold growth, unlike wood replacements made with sawdust, says company president Jim Mahler.

Fly ash also can be combined with water and pressed into bricks that harden without the use of clay, heat, or portland cement, says Henry Liu, president of the Freight Pipeline Company in Columbia, Missouri, who invented a process for producing such bricks. Freight Pipeline has licensed its Greenest Brick technology to companies in 11 countries. U.S. licensee CalStar Products of Newark, California, plans to start producing bricks at the end of 2009 near a Wisconsin coal-fired power plant run by We Energies. CalStar will be capable of making 40 million bricks a year, says chief operating officer Tom Pounds.

“People are coming to realize that when you build or renovate a building you are laying down a huge carbon footprint from the energy required to make the materials,” says Pounds. Largely due to the 1,100°C heating needed to convert clay into brick, he says, “The embodied energy in a single clay brick is about 6,000 BTUs, and we expect the fly ash bricks to be well under 1,000 BTUs.”

Another major application of CCW is the utilization of flue gas desulfurization (FGD) waste in wallboard. Power plants often remove sulfur oxides from their emissions by using “scrubbers” that spray powdered limestone into the coal smoke. A chemical reaction creates calcium sulfite, which can be oxidized into calcium sulfate—a synthetic counterpart to the gypsum rock used in wallboard. Approximately 33% of the gypsum that was used to make U.S. wallboard in 2008 was FGD gypsum, says Michael Gardner, executive director of the Gypsum Association, a trade group, who adds, “Only cutbacks in construction due to the recession have prevented the use of even more FGD gypsum.”

## Playing It Safe: Construction Materials and Leaching

The heat of coal combustion eliminates compounds such as dioxins and polycyclic aromatic hydrocarbons that could form during combustion, according to “PAHs and Dioxins Not Present in Fly Ash at Levels of Concern,” a presentation by Lisa Bradley and colleagues at the 2009 World of Coal Ash meeting, a biennial conference organized by the ACAA and the University of Kentucky Center for Applied Energy Research. But CCW can contain concentrated amounts of many other toxics that occur naturally in coal, including arsenic, mercury, boron, cadmium, and chromium. Skeptics of CCW say these toxics may leach from many “beneficial uses,” such as minefills or embankments. Can they leach from construction materials as well?

For safety purposes, LifeTime Composites tests fly ash before using it in its LifeTime Lumber product. “We do not want to run the risk of having a product that exceeds limits [for heavy metals] in our system,” says Mahler. “Our process encapsulates the ash [in polyurethane] to the point where no heavy metals are released in any way to humans, pets, or plants.”

At the 2007 World of Coal Ash meeting Liu reported on a test simulation of heavy rain at a construction site where Greenest Bricks were stored. “We compared the water sample to the EPA standard for drinking water, and every item—lead, selenium, and so on—was 10, 100, or 1,000 times less than the standard,” he says.

Meanwhile, Pounds says CalStar has submitted its products to all relevant U.S. and California EPA tests for leaching and surface wipe tests. These tests were managed and reviewed by Massachusetts-based consultancy Gradient Corp., which concluded, “[T]he presence of [CCW] metals in newly manufactured CalStar bricks is not expected to result in any exposures of health concern via dermal contact with brick surfaces or via leaching.” Gradient’s report, including the test data, is available on CalStar’s website at http://www.calstarproducts.com/.

Like the natural rock, FGD gypsum contains heavy metals. In tables prepared to accompany its March 2008 brochure “Agricultural Uses for Flue Gas Desulfurization (FGD) Gypsum,” the EPA shows higher levels of antimony, arsenic, and mercury in FGD gypsum than in natural gypsum, although in every case except that of selenium and thallium, metal levels in natural and FGD gypsum overlapped or fell below national average background levels in soil. U.S.-made dry-wall containing FGD gypsum has been tested by time, says Gardner: “We have two decades of history that have shown no adverse effects.”

However, the high temperatures involved in the production of wallboard from FGD gypsum (as well as in cement manufacturing) can cause the release of mercury, according to an article by Constance L. Senior and colleagues published in the July 2009 issue of the Air & Waste Management Association’s *EM* magazine. “The wide variation in mercury loss (2 to 55%) from seven FGD gypsum samples [taken from five plants] was attributed to the different conditions under which each gypsum sample was generated,” the authors wrote. “Any remaining mercury in the finished FGD-wallboard could be released during use or subsequent disposal or recycling of the wallboard.” The authors noted that research is under way at the EPA to evaluate the fate of mercury and other metals through each stage of wallboard’s life cycle.

Heavy metals tend to stay put in conventional concrete, says Kosmatka, who cites a 2007 PCA-financed study of concrete that passed the EPA’s toxicity characteristic leaching procedure (TCLP) test despite containing cement carrying up to 0.1% lead, cadmium, and chromium. The study, titled *Comparison of Mortar Leaching Methods*, concluded that cement containing less than 500 mg/kg of these elements would even be usable in drinking water systems.

Fly ash has been present in other studies of concrete leaching. A 1993 PCA review on the stability of concrete products, titled *Leaching of Trace Metals from Concrete*, reported on a simulation of how acid rain would affect concrete made with CCW. The concentrations of lead and cadmium from the leachates “were lower than the detection limits of the test”—that is, 0.026 ppm for cadmium and 0.125 ppm for lead. However, points out Lisa Evans, an attorney for the advocacy group Earthjustice, the detection limit for both these metals is above EPA limits for these contaminants in drinking water, which are 0.005 ppm for cadmium and 0.015 ppm for lead.

Concrete is especially likely to release toxic components shortly after mixing, during the curing and hardening phase. Harold Walker, an associate professor of civil and environmental engineering at The Ohio State University, measured the release of airborne mercury while concrete containing fly ash cured for 28 days. “Less than 0.022% of the total quantity of mercury present from all mercury sources in the concrete was released during the curing process,” Walker and colleagues wrote in volume 23, issue 4 (2009) of *Energy and Fuels*, “and therefore, nearly all of the mercury was retained in the concrete.” The authors noted that the addition of powdered activated carbon appeared to play an important role in reducing the total amount of mercury released. They also pointed out that their calculations did not address the potential release of mercury if the concrete were eventually crushed and landfilled.

In 2008, Chin-Min Cheng, then a Ph.D. student at The Ohio State University under Walker’s supervision, simulated 20 years of traffic on pavement made with concrete in which the portland cement was cut with 0%, 30%, or 50% fly ash. Leaching of 22 elements, including mercury, lead, and cadmium, was comparable from the portland cements that contained 0% or 30% fly ash, and slightly higher in the 50% blend (a level seldom used in road concrete). “The incorporation of fly ash . . . resulted in little or no deleterious environmental impact from the leaching of inorganic elements over the lifetime of the pavement system,” Cheng and colleagues wrote in the August 2008 *Journal of Environmental Engineering*.

The degree of heavy metal leaching from highway applications “depends on the chemical character of the ash, the hydrologic setting, and whether the surface will be concrete or asphalt,” Benson says. “In almost all cases, the heavy metals get bound up with minerals after they move out of the CCW layer [and into soil or subsoil].”

## Impacts of Regulation

The EPA requirement that many electric generators remove mercury from their chimney emissions poses twin challenges for fly ash recycling. First, depending on the mercury removal technique used, the amount of mercury in the fly ash rises by up to 184 times, according to tests reported by Amy Dahl of Frontier GeoSciences at the 2008 MEGA Symposium, a meeting sponsored by the EPA, the Department of Energy, the Electric Power Research Institute, and the Air & Waste Management Association. One way to avoid an increase in mercury in the fly ash is to collect the fly ash before the mercury is extracted from the flue gas stream, says Robert Meidl, a senior environmental engineer at We Energies, a Midwestern utility.

**Is minefilling really “beneficial”?**

Although use of CCW in construction materials appears to be largely safe as far as leaching of toxic ash constituents goes, not all recycling uses touted as “beneficial” are necessarily as low-risk, as illustrated by this depiction of a typical solid waste landfill compared with the use of CCW as minefill.

Second, the activated carbon that is typically used to capture mercury from emissions can inhibit the air-entraining compounds that enable concrete to resist freeze/thaw cycles. To work around this problem, Sid Nelson, global business director for mercury controls at Albemarle Sorbent Technologies Corp., says his company has developed and applied for patents on a mercury adsorbent that contains bromine. During a full-scale test at a Chicago utility that was reported at the 2007 World of Coal Ash meeting, Nelson says 80–90% of the mercury was removed, causing a 10-fold rise in the mercury content of the ash. Tests showed the ash was usable in concrete with the addition of more air-entraining additive.

The question this raises is whether these increased levels of mercury in fly ash would change any of the leaching test results referenced above, says Pounds. “This is not yet well understood, to our knowledge. In any case, it points to the need for thoughtful regulatory approaches by EPA and others that would ideally reward utilities that take [steps] to preserve their ash rather than contaminate it.”

A massive December 2008 coal ash spill in Tennessee has renewed calls that CCW be regulated as hazardous waste under Subtitle C of the Resource Conservation and Recovery Act, which is seen by some as essentially the only way the federal government can regulate disposal under existing laws. The EPA has twice considered and declined to make this designation, which many industry observers say would be a death knell to CCW recycling. “We think the stigma of being designated as hazardous would severely cripple or destroy beneficial use, simply because when the public has the option of using a material that is hazardous versus nonhazardous, [the nonhazardous option wins],” says Tom Adams, executive director of the ACAA.

Evans, a former EPA official who has testified before Congress in favor of some types of CCW recycling, favors a middle-ground option: designating the waste as hazardous when it is disposed but not when recycled into certain products, including cement and wallboard. Smaller-scale precedents for such a decision exist, she says, as in the recycling of zinc-containing waste from steel mills.

But Adams argues such a compromise might fail. “The engineers who design structures, the producers who make concrete, and the contractors who pour concrete tell us they will not expose themselves to the potential litigation they would face for building with something that was designated as hazardous. Some utilities have told us that . . . they would have to put 100% into disposal. They could not take the chance of putting some into the marketplace and facing some creative litigation.” [For more information about the debate over CCW regulation, see “Balancing Act: Creating the Right Regulation for Coal Combustion Waste,” p. A498 this issue.]

Decisions about handling CCW pose a tortuous set of tradeoffs. Coal burning has come under attack for greenhouse gas releases and for the environmental damage resulting from mining and CCW disposal. Recycling can attenuate the overall greenhouse impact and the hazards associated with ash storage, but millions of tons of CCW are now put to “beneficial uses” that, absent adequate monitoring, raise environmental questions. Increased recycling may reduce more questionable forms of “beneficial use” along with the risks and environmental costs of waste disposal.

But the prospect of increasing the recycling of CCW alarms some environmentalists who favor a move away from coal. They believe increased recycling constitutes an implicit endorsement of coal use and lessens the urgency to find alternative sources of energy.

Pounds takes another view: “The coal will be consumed to produce power regardless of the end use or designation of the [CCW]—and the challenge of replacing coal power with cleaner alternatives will remain urgent. The alternative—to simply landfill fly ash and to not take the significant environmental benefits from substituting fly ash for cement and other applications—would be irresponsible.”

**What about the CCW that isn’t recycled?**

In 2007, about 75 million tons of CCW that was not put to use went to landfills and impoundments, according to the American Coal Ash Association, raising the potential for groundwater pollution and ash spills like the one at Kingston, Tennessee, in 2008. In September 2009, in response to Freedom of Information Act requests, the U.S. EPA released a spreadsheet to Earthjustice listing 29,350 acres of impoundments that contain liquid coal waste and may still be receiving waste. The actual disposal footprint must be much larger, says Earthjustice attorney Lisa Evans, because the spreadsheet excluded landfills and older impoundments that have dried out, and the area of 74 impoundments was withheld as “confidential business information.”

Evans makes a similar point. “The increased disposal costs brought by national minimum standards for coal ash landfills and closure of unsafe coal ash impoundments will greatly increase the incentive for power plants to recycle, not dispose of the wastes,” she says. “Federal regulations that require greater scrutiny and monitoring of beneficial reuse applications will go a long way toward promoting safe recycling and eliminating the reuses that pose serious threats to health and the environment.”

## Figures and Tables

**Figure:**
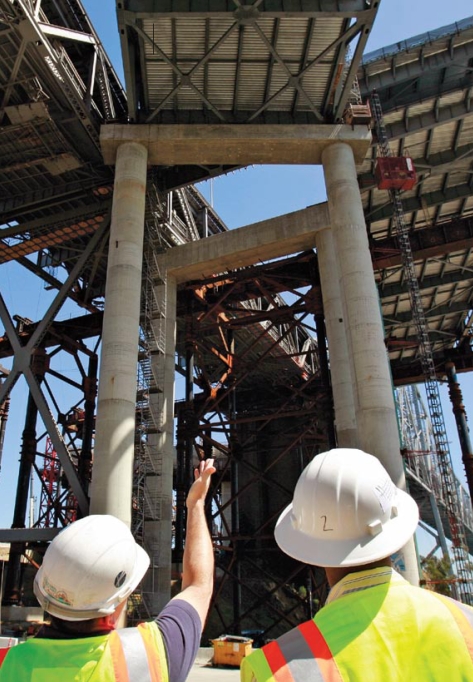
A retrofit of the San Francisco Oakland Bay Bridge involves more than 30 different concrete mixes, each of which confers specific advantages. For instance, according to the U.S. EPA, concrete containing more than 50% fly ash resists the cracking and corrosion associated with seawater. Reuters/Robert Galbraith

**Figure:**
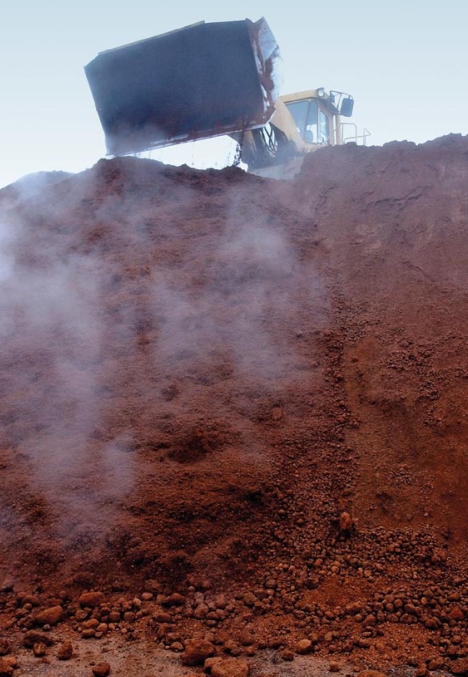
The Springdale Pit, a former surface mine in Tamaqua, Pennsylvania, is used to store coal combustion waste produced by power plants in Pennsylvania, New Jersey, and New York. When states rule that minefilling is a “beneficial use” of CCW, it is exempt from environmental safeguards that would be applied to disposal sites. AP Photo/Carolyn Kaster

**Figure:**
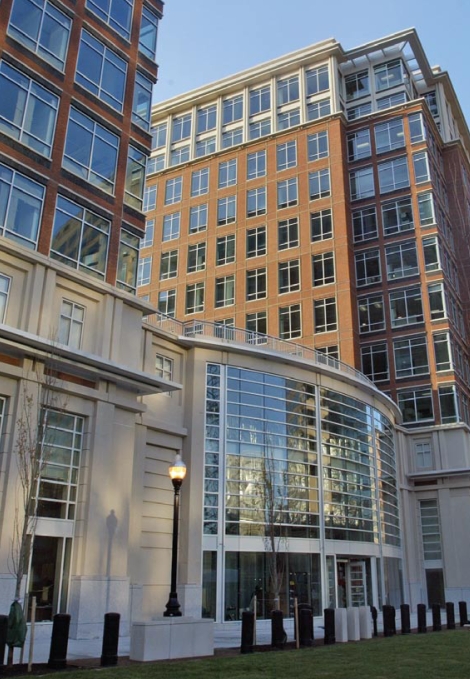
The EPA buildings at One and Two Potomac Yard in Arlington, Virginia, received LEED^®^ (Leadership in Energy and Environmental Design) Gold certification, in part for their use of environmentally preferable building materials, many of which contain CCW. Steven King

**Figure:**
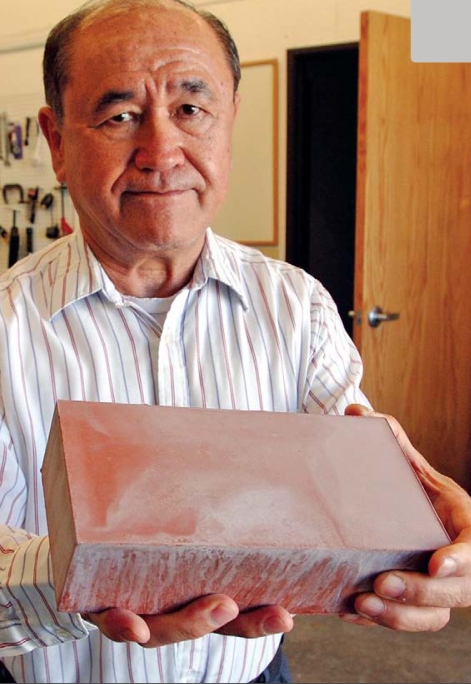
Henry Liu, president of Freight Pipeline Company, holds his company’s Greenest Brick. Unlike conventional clay bricks, these fly ash bricks harden without baking. Testing so far indicates the bricks do not pose a human health threat through leaching or dermal contact. AP Photo/L.G. Patterson

**Figure:**
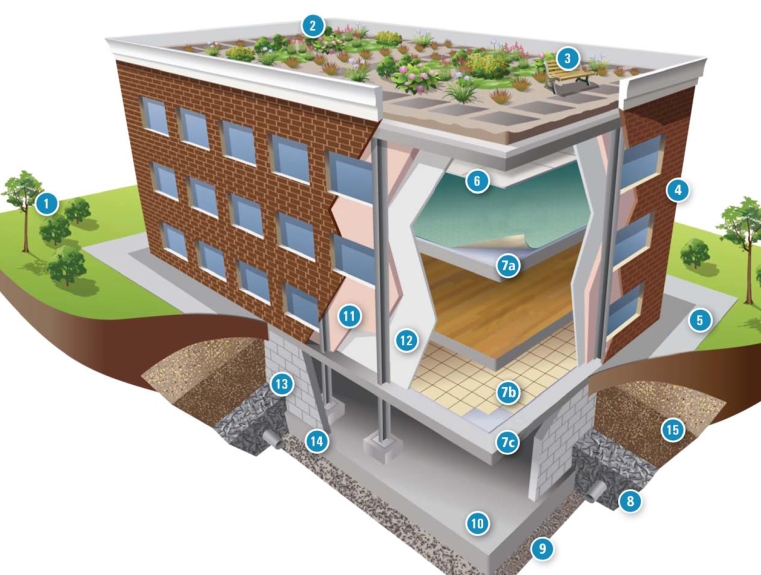
**Use of CCW in buildings** **1 & 2 Green roof & landscaping** Green roofs covered with plants reduce storm runoff and provide insulation. Bottom ash is used as a bedding material, and FGD materials and fly ash are used as soil amendments. **3 Outdoor furniture** Benches can be made with manufactured lumber containing fly ash. **4 Building facing material** Fly ash can be used in the production of bricks and other manufactured stone. **5 Sidewalk** Concrete is composed of portland cement, aggregate (sand and/or rock), and water. Fly ash added to concrete can increase durability. **6 Ceiling tile** Ceiling tile can contain FGD gypsum and fly ash. **7a Carpet backing** Carpet backing may be made with fly ash. **7b & 7c Flooring tile & tile underlayment** Flooring tile and tile underlayment may be made with fly ash. **8 Backfill (foundation support)** Backfill surrounds the building foundation, supporting it and providing drainage. Recycled concrete, which may contain fly ash, can be used for drainage. **9 Foundation structural fill** Structural fill is constructed in layers and compacted to a desired density. Fly ash, bottom ash, and boiler slag all can be used as structural fill. Recycled concrete also can be crushed and used as structural fill. **10 Poured concrete foundation** Concrete is used in a wide array of building applications, inside and out. Fly ash can partially replace portland cement, portland cement itself can be made with fly ash and FGD gypsum, and concrete aggregates can include bottom ash and recycled concrete. **12 Interior wall** FGD gypsum is used to manufacture wallboard. **13 Mortar, grout, & stucco** Fly ash can partially replace the portland cement in mortar, grout, and stucco. **14 Masonry block** Masonry blocks are made from cement and aggregate. Fly ash can partially replace portland cement, while bottom ash and recycled concrete can substitute for virgin aggregate. **15 Base material** Recycled concrete is commonly used as a base material. Adapted from U.S. EPA. Using recycled industrial materials in buildings. EPA-530-F-08-022. Washington, DC: U.S. Environmental Protection Agency; 2008: p. 2–3.

**Figure:**
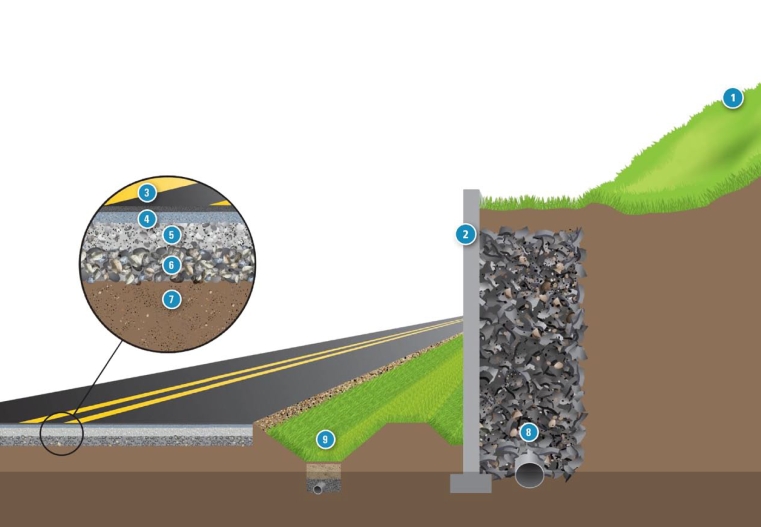
**Use of CCW in roadways** **1 Embankment** Topsoil on roadside embankments can be amended with FGD materials if soil conditions permit. FGD materials can improve the condition of the soil, increase plant growth, and reduce runoff. Coal ash is suitable for embankment fill. **2 Retaining wall** Retaining walls hold back soil and rock and prevent the erosion of roadside slopes; they are often made of concrete or modular blocks. Fly ash can partially replace portland cement in concrete, making the concrete stronger and more durable. Portland cement also can contain FGD gypsum. Concrete aggregates can include bottom ash and recycled concrete, which may contain fly ash. **3 Asphalt surface** Boiler slag can replace virgin aggregate in the asphalt surface layer. **4 Asphalt base** Fly ash, bottom ash, and recycled concrete can be used as aggregate in the asphalt base layer. **5 & 6 Granular base & sub-base** A variety of industrial materials can be used as granular base and sub-base, including bottom ash and recycled concrete. Fly ash also can be used as mineral filler in asphalt base, granular base, and sub-base. **7 Subgrade (original soil)** Fly ash can improve the structure and stability of the subgrade upon which the road will be built. **8 Structural fill** Structural fill supports and relieves pressure from retaining walls. Fly ash and recycled concrete can be used as backfill for retaining walls. **9 Vegetated swale** Vegetated swales provide drainage for roadways and help improve water quality. Recycled concrete can be used in place of traditional drainage materials, such as virgin sand or gravel. Adapted from U.S. EPA. Using recycled industrial materials in roadways. EPA-530-F-08-024. Washington, DC: U.S. Environmental Protection Agency; 2009: p. 2–3.

**Figure:**
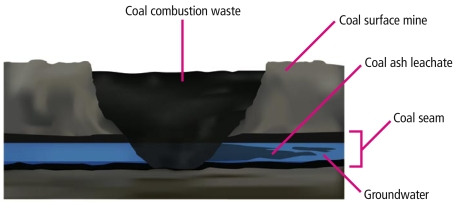
Coal Combustion Waste Minefill Matt Lau/Earthjustice

**Figure:**
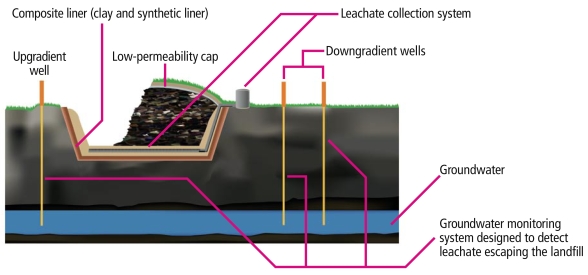
Solid Waste Landfill

